# Kinetics of Phosphomevalonate Kinase from *Saccharomyces cerevisiae*

**DOI:** 10.1371/journal.pone.0087112

**Published:** 2014-01-27

**Authors:** David E. Garcia, Jay D. Keasling

**Affiliations:** 1 Joint BioEnergy Institute, Emeryville, California, United States of America; 2 Department of Chemistry, University of California, Berkeley, California, United States of America; 3 Physical Biosciences Division, Lawrence Berkeley National Laboratory, Berkeley, California, United States of America; 4 Department of Chemical & Biomolecular Engineering, University of California, Berkeley, California, United States of America; 5 Department of Bioengineering, University of California, Berkeley, California, United States of America; National Centre for Cell Science, India

## Abstract

The mevalonate-based isoprenoid biosynthetic pathway is responsible for producing cholesterol in humans and is used commercially to produce drugs, chemicals, and fuels. Heterologous expression of this pathway in *Escherichia coli* has enabled high-level production of the antimalarial drug artemisinin and the proposed biofuel bisabolane. Understanding the kinetics of the enzymes in the biosynthetic pathway is critical to optimize the pathway for high flux. We have characterized the kinetic parameters of phosphomevalonate kinase (PMK, EC 2.7.4.2) from *Saccharomyces cerevisiae*, a previously unstudied enzyme. An *E. coli* codon-optimized version of the *S. cerevisiae* gene was cloned into pET-52b+, then the C-terminal 6X His-tagged protein was expressed in *E. coli* BL21(DE3) and purified on a Ni^2+^ column. The K_M_ of the ATP binding site was determined to be 98.3 µM at 30°C, the optimal growth temperature for *S. cerevisiae*, and 74.3 µM at 37°C, the optimal growth temperature for *E. coli*. The K_M_ of the mevalonate-5-phosphate binding site was determined to be 885 µM at 30°C and 880 µM at 37°C. The V_max_ was determined to be 4.51 µmol/min/mg enzyme at 30°C and 5.33 µmol/min/mg enzyme at 37°C. PMK is Mg^2+^ dependent, with maximal activity achieved at concentrations of 10 mM or greater. Maximum activity was observed at pH = 7.2. PMK was not found to be substrate inhibited, nor feedback inhibited by FPP at concentrations up to 10 µM FPP.

## Introduction

The mevalonate pathway is an important conduit for the production of crucial metabolites with a wide array of functions, including terpenoids [1], [2], hormones and steroids [3]. The heterologous expression of this pathway in *Escherichia coli* has enabled high-level production of the antimalarial drug artemisinin [4]–[6], but the chemical structures of these metabolites also make them interesting targets for solving some of the most crucial problems in the energy market [7], [8]. With only slight modifications to mevalonate pathway intermediates and products, either *in vivo* or through traditional chemical engineering processes post cell culture extraction, these molecules can be transformed into biofuels that, depending on our ability to scale-up, could offset or replace traditional liquid fuels [9]. This would allow us to replace petroleum-based, CO_2_ producing fuels with fuels that are carbon neutral. Although industrial-scale corn-based ethanol production is already a reality in the energy market, ethanol is a less than desirable biofuel because not only does it divert crops from the food supply, it is not compatible with our current distribution infrastructure or vehicle fleet [10].

Whether these fuel alternatives are five-carbon alcohols derived from the mevalonate pathway intermediates isopentenyl pyrophosphate and dimethylallyl pyrophosphate [11], or downstream, terpene-based molecules like bisabolene [8], further improvement of titers may be realized through a more robust understanding of the enzymes in the mevalonate pathway and the ways in which those enzymes are regulated by metabolic intermediates. In particular, proteomics data has previously shown that the fourth and fifth enzymes in the pathway—mevalonate kinase (MK) and phosphomevalonate kinase (PMK), respectively—are expressed at relatively low levels and may be targets for increasing overall isoprenoid production [12], [13]. Previous work has also shown that substrate inhibition and feedback inhibition of MK may be responsible for limiting flux through the pathway [14]. Because MK—a phosphotransferase that acts on mevalonate and ATP to yield mevalonate-5-phosphate—and PMK—a phosphotransferase that acts on mevalonate-5-phosphate and ATP to yield mevalonate-5-diphosphate—both require ATP to function and downstream prenyl phosphates might act as general ATP binding site inhibitors, PMK was identified as another potential source of pathway regulation.

PMKs from other sources have been studied revealing implications for pathway engineering. For example, PMK from *E. faecalis* is Mn^2+^ dependent rather than Mg^2+^ dependent [15]. Pig-derived PMK is substrate inhibited by ATP under high ATP, low mevalonate phosphate concentrations [16]. If *S. cerevisiae* PMK is similarly dependent or inhibited it would make an ideal target for protein engineering. Furthermore, *S. cerevisiae* prefers to grow at 30°C, but much of our production takes place in *E. coli*, which necessitates understanding how PMK activity is affected by a change in growth temperature from 30°C to 37°C. Herein we report cloning a codon-optimized sequence of *S. cerevisiae* PMK into an expression vector, the expression and purification of PMK in *E. coli*, and the kinetic characterization of the purified enzyme.

## Results and Discussion

**Figure 1:**
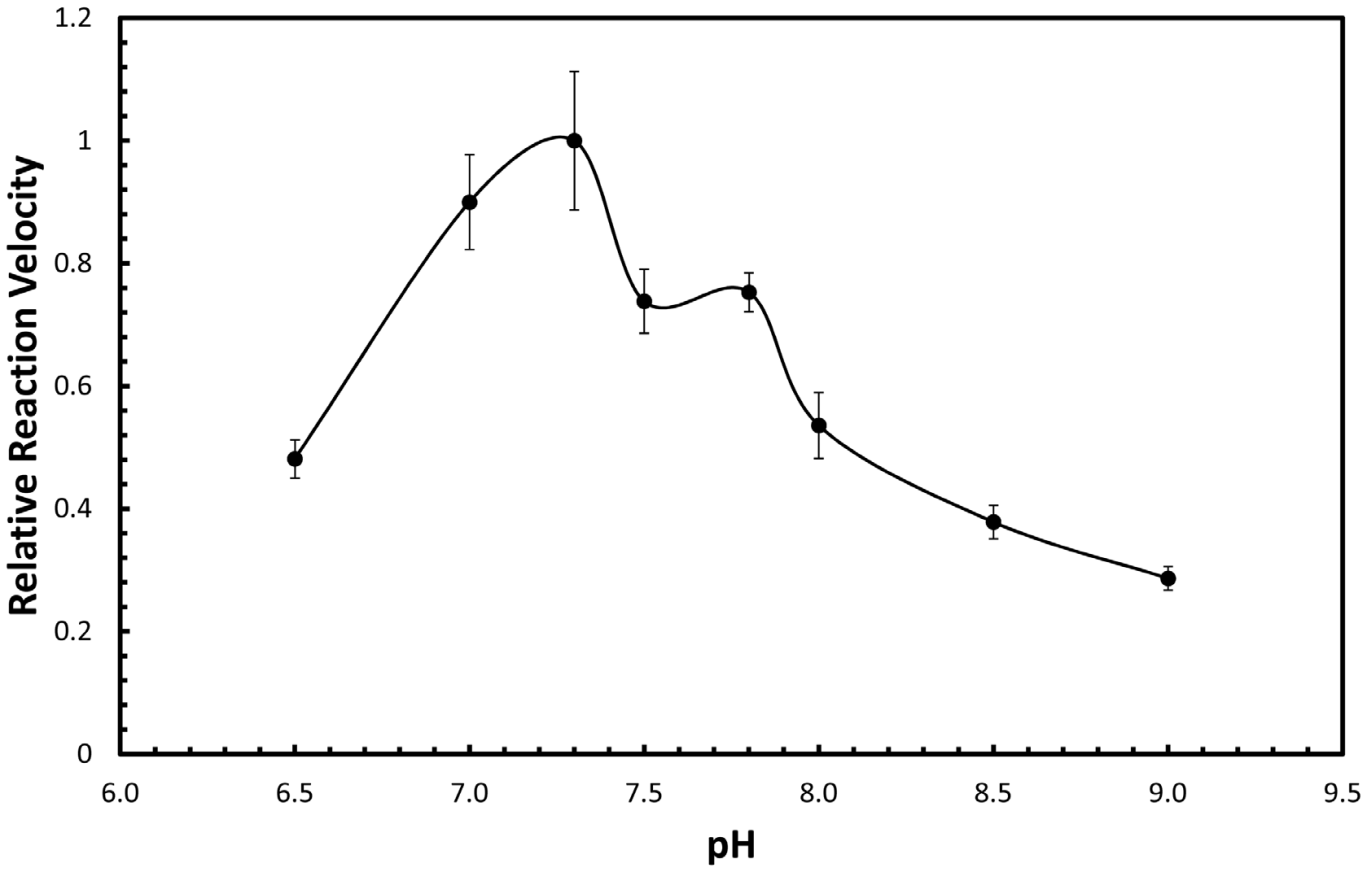
pH dependence of *S. cerevisiae* phosphomevalonate kinase.

**Figure 2:**
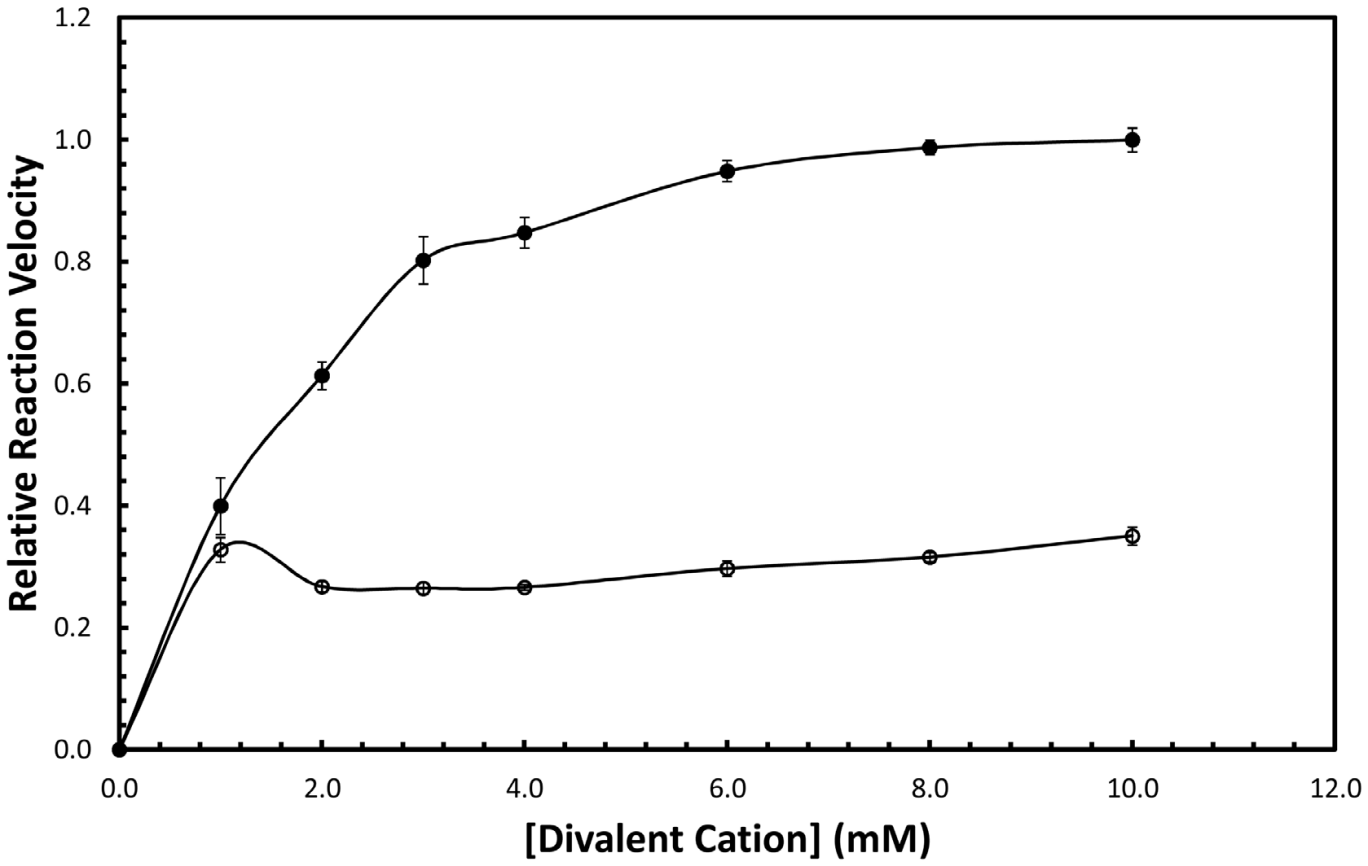
Divalent cation dependence. Closed circles are data for Mg^2+^ and open circles are data for Mn^2+^.

Although phosphomevalonate kinase (PMK) from *S. cerevisiae* has previously been studied in partially purified lysates [17], and even utilized to study the kinetics of another enzyme [18], this is the first time PMK from *S. cerevisiae* has been kinetically characterized in isolation. In a study of the partially purified enzyme it was reported that pH did not affect PMK activity, but we found that PMK does have an optimal activity at pH = 7.2, and its activity drops off below pH = 6.5 and above pH = 8.0 (Figure 1). Although at first glance there is an apparent “shoulder” in the pH profile, careful consideration of the profile shows that the shoulder is within error and therefore cannot be considered to conclusively exist. Although we did not test a wide array of storage conditions, solutions with high PMK concentrations were found to be stable long term only at pH = 8.0 with 800 mM NaCl. As found previously *S. cerevisiae* PMK shows a cation dependence on Mg^2+^, with 10 mM corresponding to maximal activity (Figure 2).

**Figure 3:**
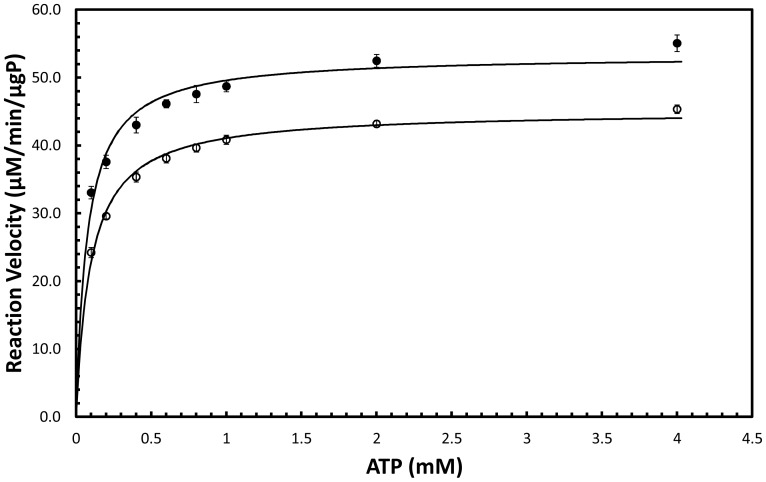
Initial reaction velocity as a function of ATP concentration. Closed circles are data for incubation at 37°C and open circles are data for incubation at 30°C.

**Figure 4:**
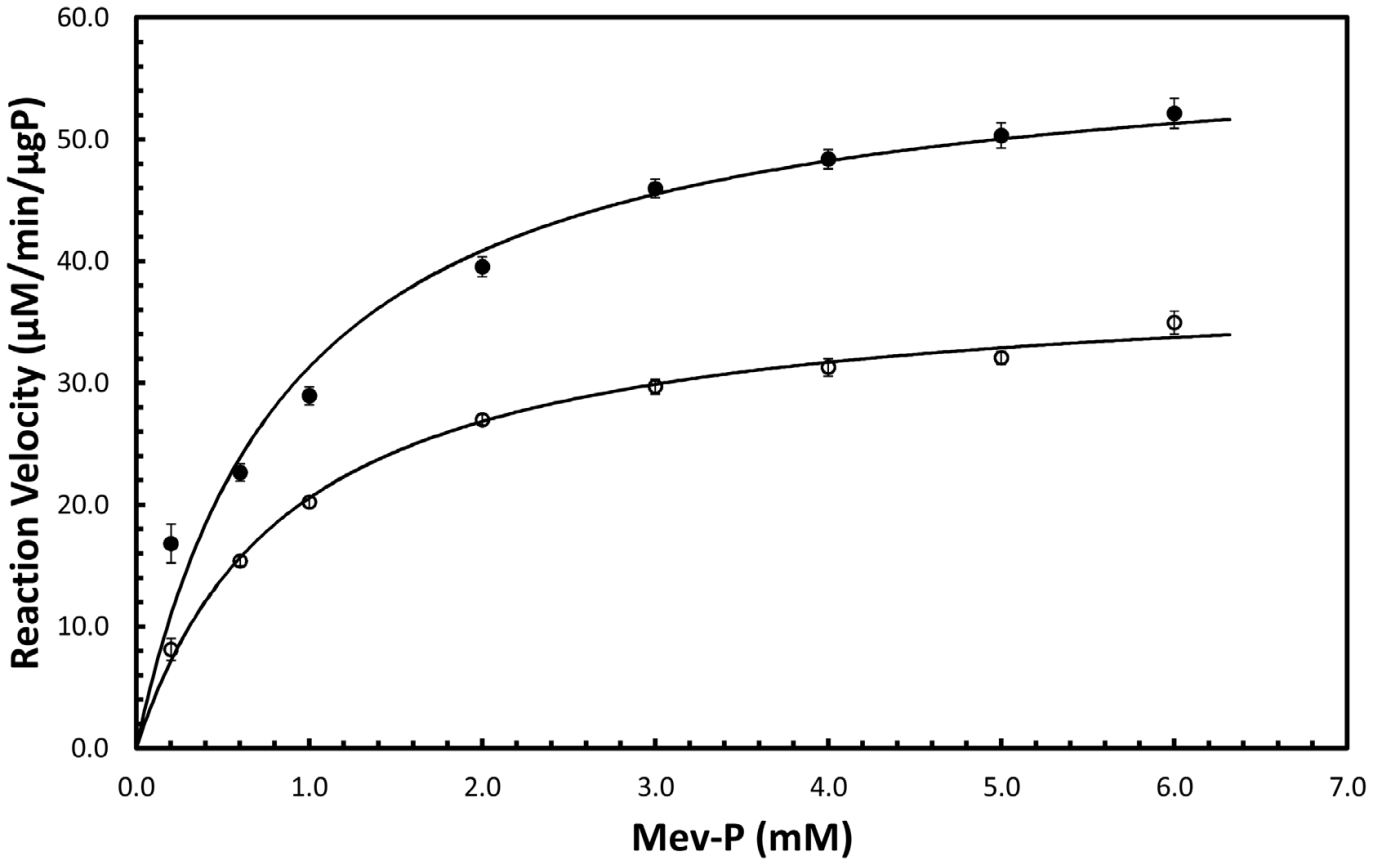
Initial reaction velocity as a function of mevalonate-5-phosphate concentration. Closed circles are data for incubation at 37°C and open circles are data for incubation at 30°C.

Kinetic constants were determined by nonlinear regression analysis using the solver function in Microsoft Excel. The K_M_ for ATP, K_M_^ATP^, was determined to be 98.3 µM and 74.3 µM at 30°C and 37°C, respectively. The K_M_ for mevalonate-5-phosphate, K_M_^mev-p^, was determined to be 885 µM and 880 µM at 30°C and 37°C, respectively (Figure 3). V_max_ was determined to be 4.51 µmol/min/µg enzyme and 5.33 µmol/min/µg enzyme at 30°C and 37°C, respectively (Figure 4). In contrast, the K_M_^ATP^, K_M_^mev-p^, and V_max_ for the *Enterococcus faecalis* PMK, which is Mn dependent, were reported to be 170 µM, 190 µM, and 3.9 µmol/min/mg enzyme [15]. The values for the *Streptococcus pneumonia* PMK were reported to be 74 µM, 4.2 µM, and 5.5 µmol/min/mg enzyme [19]. The values for pig liver PMK have been reported to be 43 µM, 12 µM, and 51 µmol/min/mg enzyme [16]. For the recombinant human PMK, the values were reported to be 107 µM, 34 µM, and 46 µmol/min/mg enzyme [20]. The high K_M_^mev-p^ for the *S. cerevisiae* PMK makes it less ideal than enzymes with a low K_M_, as it would only reach its maximal rate at a high concentration of mevalonate-5-phosphate. Because of the Mn dependence of the *E. faecalis* PMK, it may not function fully if expressed in *E. coli* or other organisms. In contrast, the *S. pneumonia,* pig, and human PMKs have reasonable values for K_M_^ATP^ and K_M_^mev-p^, making them better choices for a heterologous pathway. In terms of maximum rates, the mammalian enzymes are high than the microbial enzymes.

Because the *S. cerevisiae* PMK has been used heterologously in *E. coli* for production of isoprenoids [4]–[8], [11], the temperature effect on PMK activity is important, particularly at *E. coli*'s optimal growth temperature of 37°C. Despite expectations that PMK activity might diminish with increasing the temperature from the preferred 30°C growth temperature of *S. cerevisiae* to the 37°C preferred by *E. coli*, PMK activity was shown to slightly increase with the increase in temperature. This increased activity bodes well for the production of isoprenoid products, including advanced biofuels, via the mevalonate pathway if the low protein expression levels currently observed can be increased [12], [13]_ENREF_9. It should be noted that although we were able to achieve very high yields of PMK using pET-52b+ for the purpose of isolating and purifying the enzyme, increasing PMK expression in production strains by using high copy plasmids would be counterproductive to increasing overall biofuels production as doing so would divert an unnecessary amount of resources into the production of protein to the detriment of fuel titers.

One regulatory mechanism for controlling PMK activity we can rule out is feedback inhibition, as the presence of farnesyl pyrophosphate (FPP)—a known inhibitor of MK [18]—did not affect PMK activity at concentrations up to 10 µM FPP (data not shown). Of the publications reporting the kinetics of PMKs from various organisms, none have reported inhibition by prenyl phosphates. Furthermore, unlike *S. cerevisiae* mevalonate kinase [18], PMK did not demonstrate substrate inhibition. The lack of feedback and substrate inhibition in the *S. cerevisiae* PMK is an attractive feature for increasing production of a desired isoprenoid. Nevertheless, *S. pneumonia* PMK, which has a high V_max_ and low K_M_s, is a much better enzyme and should be incorporated into future production strains. An additional advantage of the *S. pneumonia* PMK is that its crystal structure of the has been solved [21] and the kinetic mechanism of its catalysis has been described in detail [19].

With the addition of PMK from this study, the *S. cerevisiae*-derived mevalonate pathway enzymes that have been kinetically characterized include hydroxymethylglutaryl synthase [22], hydroxymethylglutaryl reductase [14], mevalonate kinase [18], phosphomevalonate decarboxylase [23], and farnesyl pyrophosphate synthase [24], leaving acetyl-CoA C-acetyltransferase and isopentenyl diphosphate isomerase uncharacterized. Although isopentenyl diphosphate isomerase has been isolated and studied [25], the difficulty associated with detecting the isomerization of a single bond is likely why the kinetic constants have yet to be determined. In combination with traditional genetic engineering techniques, such as varying promoter strength, and newly developed technologies for varying expression, such as RBS calculators [26], studying the kinetics of these remaining enzymes should allow isoprenoid production from engineered microbes to be optimized more rationally.

## Materials and Methods

### Codon Optimization of PMK

The original *S. cerevisiae* PMK sequence (accession number NM_001182727), which was downloaded from the BioCyc.org database, was codon optimized by DNA2.0 (Menlo Park, CA) for expression in *E. coli*. Codon optimization replaced codons rare for *E. coli* with more frequently used codons. The sequences of the original and codon-optimized versions of the genes are presented in Figure S1.

### Expression Plasmid Construction

A chemically-competent strain of *E. coli* DH10B was transformed with pET-52b+ (Novagen, Germany) and then used to prepare the plasmid according to the instructions and materials in a Qiagen (Valencia, California) Spin Miniprep Kit. The codon-optimized PMK sequence was PCR amplified with primers that added a BsaI restriction site with an *Nco*I overhang on the 5′ end of the sequence and a *Sac*I restriction site on the 3′ end of the sequence, then digested with the appropriate restriction enzymes (all enzymes from New England BioLabs, Ipswich, Massachusetts), and cloned into pET-52b+ to make expression plasmid pET-52b+_coPMK-His. Confirmation of expression plasmid construction was accomplished by sequencing the cloning region using T7 primers (sequencing and primers from Quintara Biosciences, Albany, California).

### PMK-His Expression and Purification

Ideal conditions for PMK expression were screened on NuPAGE 10% Bis-Tris SDS-PAGE gels and the supplies indicated in the accompanying protocol (Invitrogen, Grand Island, New York) from 5-mL cultures that spanned a range of media types, growth temperatures, inducer concentrations, and growth times. Protein expression was ultimately accomplished by growing a 2-L culture in Terrific Broth (Invitrogen) to OD_600_ = 0.6 at 37°C, inducing with 100 µM IPTG (Sigma Aldrich, St. Louis, Missouri), then growing at 18°C for approximately two days (until stationary phase was reached). Cells were pelleted in 250-mL portions, flash frozen in liquid nitrogen after medium removal, and then stored at −80°C prior to further processing. On ice, cells from one 250-mL portion were suspended in 25 mL of a lysis buffer (10 mM Imidizole, 300 mM NaCl, 50 mM NaH_2_PO_4_, pH = 8.0; Sigma Aldrich), sonicated for 10 minutes in a water bath to break up residual clumps, then homogenized with two passes through an EmulsiFlex®-C3 (Avestin, Canada). Cell debris was removed by centrifugation at 12,000 X g for 30 minutes. Cleared lysate was bound to 2-mL of Ni-NTA resin (Qiagen) at 4°C by rocking gently for 30 minutes. The resin was then bedded in a column, washed with 20 column volumes (CV) of buffer containing 20 mM imidizole, then the protein was eluted with 10 CV of buffer containing 500 mM imidizole. Buffer exchange into 20 mM Tris, 50 mM NaCl, pH = 7.0 was accomplished on an AKTA (GE Healthcare Life Sciences, Pittsburgh, Pennsylvania) using a GE Healthcare HiPrep 26/10 Desalting Column (17-5087-01). Protein was then concentrated using VivaSpin 20 3,000-MWCO filters (Sartorius, Bohemia, New York). Protein concentration was determined using a Nanodrop (Thermo Scientific, West Palm Beach, Florida). The protein was then diluted so that glycerol (Sigma) was 50% v/v and stored at −20°C.

### Activity Assay

All chemicals and supporting enzymes were purchased from Sigma-Aldrich. Reaction progress was monitored spectrophotometrically at 339 nm for NADH consumption on a 96-well plate in a Spectramax M2 (Molecular Devices, Sunnyvale, California). 100-µL enzymatic assay mixtures contained 200 mM Tris (pH = 7.2), 100 mM KCl, 10 mM MgCl_2_, 0.81 mM NADH, 1.5 mM phosphoenolpyruvate, 0.682U pyruvate kinase, 0.990 U lactate dehydrogenase, 0.1 µg PMK, 0.1–8.0 mM ATP, and 0.2–10.0 mM mevalonate-5-phosphate. Stock concentrations of NADH and pH neutralized ATP were confirmed through their extinction coefficients (^ATP^ε_259 nm_ = 15.4 mM^−1^ cm^−1^, ^NADH^ε_339 nm_ = 6.22 mM^−1^ cm^−1^). All conditions were repeated twelve times for statistical analysis, from which K_M_ (µM) and reaction velocities (µM mev-PP formed*minute^−1^ * µg PMK^−1^) were calculated. When studying pH effect and divalent cation dependence, ATP and mevalonate-5-phosphate were held constant and data were normalized to the maximum observed reaction velocities. To ensure PMK was the rate-limiting enzyme, when necessary the following standard controls and results were verified: doubling the PMK added doubled the observed rate, doubling the supporting enzymes added did not affect the observed rate, and doubling the phosphoenolpyruvate concentration did not affect the observed rate.

## Supporting Information

Figure S1 Sequences of the original PMK and the codon-optimized version of PMK.
(DOCX)
