# Supplementary material for: Kinetics of Phosphomevalonate Kinase from Saccharomyces cerevisiae
Source: PLoS One. 2014 Jan 27;9(1):e87112. doi: 10.1371/journal.pone.0087112 (PMC3903622; doi:10.1371/journal.pone.0087112)
Supplement: Figure S1 — Sequences of the original PMK and the codon-optimized version of PMK. (DOCX) [file pone.0087112.s001.docx]

**Figure S1.** Sequences of the original PMK and the codon-optimized version of PMK.

**Original PMK**

ATGTCAGAGTTGAGAGCCTTCAGTGCCCCAGGGAAAGCGTTACTAGCTGGTGGATATTTAGTTTTAGATACAAAATATGAAGCATTTGTAGTCGGATTATCGGCAAGAATGCATGCTGTAGCCCATCCTTACGGTTCATTGCAAGGGTCTGATAAGTTTGAAGTGCGTGTGAAAAGTAAACAATTTAAAGATGGGGAGTGGCTGTACCATATAAGTCCTAAAAGTGGCTTCATTCCTGTTTCGATAGGCGGATCTAAGAACCCTTTCATTGAAAAAGTTATCGCTAACGTATTTAGCTACTTTAAACCTAACATGGACGACTACTGCAATAGAAACTTGTTCGTTATTGATATTTTCTCTGATGATGCCTACCATTCTCAGGAGGATAGCGTTACCGAACATCGTGGCAACAGAAGATTGAGTTTTCATTCGCACAGAATTGAAGAAGTTCCCAAAACAGGGCTGGGCTCCTCGGCAGGTTTAGTCACAGTTTTAACTACAGCTTTGGCCTCCTTTTTTGTATCGGACCTGGAAAATAATGTAGACAAATATAGAGAAGTTATTCATAATTTAGCACAAGTTGCTCATTGTCAAGCTCAGGGTAAAATTGGAAGCGGGTTTGATGTAGCGGCGGCAGCATATGGATCTATCAGATATAGAAGATTCCCACCCGCATTAATCTCTAATTTGCCAGATATTGGAAGTGCTACTTACGGCAGTAAACTGGCGCATTTGGTTGATGAAGAAGACTGGAATATTACGATTAAAAGTAACCATTTACCTTCGGGATTAACTTTATGGATGGGCGATATTAAGAATGGTTCAGAAACAGTAAAACTGGTCCAGAAGGTAAAAAATTGGTATGATTCGCATATGCCAGAAAGCTTGAAAATATATACAGAACTCGATCATGCAAATTCTAGATTTATGGATGGACTATCTAAACTAGATCGCTTACACGAGACTCATGACGATTACAGCGATCAGATATTTGAGTCTCTTGAGAGGAATGACTGTACCTGTCAAAAGTATCCTGAAATCACAGAAGTTAGAGATGCAGTTGCCACAATTAGACGTTCCTTTAGAAAAATAACTAAAGAATCTGGTGCCGATATCGAACCTCCCGTACAAACTAGCTTATTGGATGATTGCCAGACCTTAAAAGGAGTTCTTACTTGCTTAATACCTGGTGCTGGTGGTTATGACGCCATTGCAGTGATTACTAAGCAAGATGTTGATCTTAGGGCTCAAACCGCTAATGACAAAAGATTTTCTAAGGTTCAATGGCTGGATGTAACTCAGGCTGACTGGGGTGTTAGGAAAGAAAAAGATCCGGAAACTTATCTTGATAAATAG

**Codon-Optimized PMK**

ATGAGCGAATTACGTGCATTCAGCGCGCCAGGTAAGGCACTGCTGGCCGGTGGCTACCTGGTGTTAGACACCAAGTACGAGGCGTTCGTCGTCGGCTTATCTGCCCGTATGCATGCAGTTGCCCACCCGTATGGTAGCCTGCAGGGCTCTGACAAGTTCGAAGTGCGTGTGAAGAGCAAGCAGTTCAAGGACGGCGAGTGGCTGTACCACATTAGCCCAAAGAGCGGCTTCATCCCGGTTAGCATTGGTGGCAGCAAGAACCCATTTATCGAGAAGGTCATTGCCAACGTCTTCAGCTACTTCAAGCCGAATATGGACGATTACTGCAACCGCAACCTGTTCGTCATCGACATTTTCAGCGACGACGCGTACCACAGCCAAGAGGACTCTGTTACGGAGCATCGTGGTAACCGCCGCCTGAGCTTCCACAGCCATCGCATTGAGGAGGTGCCGAAGACGGGTCTGGGTTCTAGCGCCGGTTTAGTTACCGTCTTAACGACGGCGTTAGCGAGCTTCTTCGTGAGCGACCTGGAGAACAACGTGGACAAGTACCGCGAAGTGATTCATAACCTGGCGCAGGTGGCACATTGTCAGGCCCAAGGTAAGATTGGCTCTGGTTTTGATGTGGCAGCGGCCGCCTATGGCTCTATCCGCTATCGCCGCTTTCCGCCGGCCCTGATCAGCAATCTGCCGGACATCGGCTCTGCGACGTATGGTAGCAAACTGGCGCATCTGGTGGACGAGGAGGACTGGAACATCACCATTAAGTCTAATCACCTGCCGAGCGGCTTAACGTTATGGATGGGCGATATCAAGAACGGCAGCGAAACGGTTAAGCTGGTGCAGAAAGTGAAAAACTGGTACGACAGCCACATGCCGGAAAGCCTGAAGATTTACACGGAGCTGGACCACGCCAATAGCCGTTTCATGGATGGTCTGAGCAAGCTGGACCGCCTGCACGAAACCCACGACGACTACAGCGACCAGATCTTCGAGAGCCTGGAGCGCAATGACTGCACCTGCCAGAAGTACCCGGAGATCACGGAGGTCCGCGATGCCGTGGCAACGATTCGCCGTAGCTTCCGCAAAATTACGAAGGAGAGCGGCGCGGATATCGAACCACCGGTCCAGACGTCTCTGCTGGACGACTGTCAAACCTTAAAGGGCGTGTTAACGTGCCTGATTCCGGGCGCGGGTGGTTACGACGCCATTGCCGTCATCACGAAACAGGACGTCGATCTGCGCGCACAAACGGCCAACGACAAACGTTTCAGCAAAGTCCAATGGCTGGATGTTACGCAGGCCGACTGGGGTGTTCGCAAGGAGAAGGACCCGGAAACGTATCTGGATAAGTGA
